# Submicroscopic and Asymptomatic *Plasmodium* Parasitaemia Associated with Significant Risk of Anaemia in Papua, Indonesia

**DOI:** 10.1371/journal.pone.0165340

**Published:** 2016-10-27

**Authors:** Zuleima Pava, Faustina H. Burdam, Irene Handayuni, Leily Trianty, Retno A. S. Utami, Yusrifar Kharisma Tirta, Enny Kenangalem, Daniel Lampah, Andreas Kusuma, Grennady Wirjanata, Steven Kho, Julie A. Simpson, Sarah Auburn, Nicholas M. Douglas, Rintis Noviyanti, Nicholas M. Anstey, Jeanne R. Poespoprodjo, Jutta Marfurt, Ric N. Price

**Affiliations:** 1 Global and Tropical Health Division, Menzies School of Health Research, Charles Darwin University, Darwin, Australia; 2 Mimika District Health Authority, Timika, Papua, Indonesia; 3 Timika Malaria Research Programme, Papuan Health and Community Development Foundation, Timika, Papua, Indonesia; 4 Maternal and Child Health and Reproductive Health, Department of Public Health, Faculty of Medicine, Universitas Gadjah Mada, Yogyakarta, Indonesia; 5 Eijkman Institute for Molecular Biology, Jakarta, Indonesia; 6 Centre for Epidemiology and Biostatistics, Melbourne School of Population and Global Health, The University of Melbourne, Melbourne, Australia; 7 Division of Medicine, Christchurch Hospital, Christchurch, New Zealand; 8 Centre for Tropical Medicine and Global Health, Nuffield Department of Clinical Medicine, University of Oxford, Oxford, United Kingdom; Ehime Daigaku, JAPAN

## Abstract

Submicroscopic *Plasmodium* infections are an important parasite reservoir, but their clinical relevance is poorly defined. A cross-sectional household survey was conducted in southern Papua, Indonesia, using cluster random sampling. Data were recorded using a standardized questionnaire. Blood samples were collected for haemoglobin measurement. Plasmodium parasitaemia was determined by blood film microscopy and PCR. Between April and July 2013, 800 households and 2,830 individuals were surveyed. Peripheral parasitaemia was detected in 37.7% (968/2,567) of individuals, 36.8% (357) of whom were identified by blood film examination. Overall the prevalence of *P*. *falciparum* parasitaemia was 15.4% (396/2567) and that of *P*. *vivax* 18.3% (471/2567). In parasitaemic individuals, submicroscopic infection was significantly more likely in adults (adjusted odds ratio (AOR): 3.82 [95%CI: 2.49–5.86], *p*<0.001) compared to children, females (AOR = 1.41 [1.07–1.86], *p* = 0.013), individuals not sleeping under a bednet (AOR = 1.4 [1.0–1.8], *p* = 0.035), and being afebrile (AOR = 3.2 [1.49–6.93], *p* = 0.003). The risk of anaemia (according to WHO guidelines) was 32.8% and significantly increased in those with asymptomatic parasitaemia (AOR 2.9 [95% 2.1–4.0], *p* = 0.007), and submicroscopic *P*. *falciparum* infections (AOR 2.5 [95% 1.7–3.6], *p* = 0.002). Asymptomatic and submicroscopic infections in this area co-endemic for *P*. *falciparum* and *P*. *vivax* constitute two thirds of detectable parasitaemia and are associated with a high risk of anaemia. Novel public health strategies are needed to detect and eliminate these parasite reservoirs, for the benefit both of the patient and the community.

## Introduction

Significant gains have been made over the last decade in the control and elimination of malaria. Early diagnosis, highly effective treatment and intense vector control have led to reductions in the number of malaria cases, as well as severe disease and associated mortality [1]. In 2014, 18 countries in the Asia-Pacific committed to eliminate malaria from the region by 2030; however, the challenges to achieve this goal are immense [2]. The final stages of malaria elimination are often prolonged, with low-grade transmission sustained by a parasite reservoir present in asymptomatic individuals who may not readily engage or attend healthcare facilities to be treated. Novel strategies are needed to identify and eliminate these asymptomatic parasite reservoirs [3].

The established standard for malaria diagnosis in the field remains blood film examination. Lower limits of detection range from 10–20 parasites/μL in research settings to more than 100/μL in clinical practice [4]. Recent advances in molecular techniques have allowed the development of highly sensitive methods for detecting peripheral parasitaemia at submicroscopic levels (0.1–10 parasites/μL) [5–8]. The application of molecular approaches in surveillance surveys has resulted in an inevitable increase in the prevalence of detectable parasitaemia, often 2–10 fold higher than the prevalence obtained by conventional methods [9]. Although most of the available data on subpatency have been derived from the studies of *P*. *falciparum*, similar observations have been made for *P*. *vivax* [10, 11].

Transmission can occur from individuals with asymptomatic microscopic parasitaemia, many of whom are semi-immune and maintain low levels of parasitaemia and gametocytaemia for sustained periods of time [12]. Submicroscopic parasitaemia can also be transmitted to the mosquito vector (albeit less efficiently) and given the higher prevalence, contributes significantly to the overall infectious reservoir [13, 14].

The clinical relevance of submicroscopic infections is less clear. In cross-sectional surveys, low-level parasitaemia may represent early detection of an expanding parasite biomass that will ultimately result in clinical disease. However, repeated exposure and acquisition of partial immunity can result in persistent low-grade infection, and although these individuals may not have fever, chronic carriage can result in anaemia, bacterial co-infection and cognitive impairment [15]. In pregnancy, asymptomatic parasitaemia has also been shown to increase the risk of maternal anaemia and the delivery of low birth weight babies [16, 17]. Thus, carriage of asymptomatic parasitaemia potentially has profound implications for the health of individuals and for the control and elimination of malaria [18–20].

We conducted a large cross-sectional survey in southern Papua, Indonesia, an area co-endemic for *P*. *falciparum* and *P*. *vivax*, to identify the risk factors for microscopic and submicroscopic carriage for both species, and thereby populations at greatest risk of harbouring undetected parasitaemia by conventional microscopic surveys. We also aimed to quantify the degree of anaemia associated with asymptomatic and submicroscopic parasitaemia, to gauge the clinical relevance of these low level infections.

## Materials and Methods

### Study Site

Timika, the main city of the Mimika District, is located in southern Papua province, Indonesia. It has a population of 202,350 composed of different ethnicities broadly including Highland Papuans and Lowland Papuans, and Non-Papuans. Nearly 90% of the population lives in lowland areas where unstable malaria transmission occurs. In 2013, the annual incidence of parasitaemia was 450/1000, with *P*. *falciparum* and *P*. *vivax* causing 60% and 40% of cases, respectively [21], without significant seasonal fluctuation in incidence. Patients with uncomplicated malaria due to any *Plasmodium* species are treated with dihydroartemisinin-piperaquine (DHP) as the first-line treatment regimen.

### Cross-Sectional Survey Methods

#### Sample selection

Households were chosen by cluster random sampling. First, the four largest subdistricts were chosen purposively. Consecutively, the numbers of cluster per district was calculated according to their respective relative population. Finally, 25 houses were chosen randomly within each cluster, following WHO recommendations. Clusters constituted whole existing villages, except in Mimika Baru subdistrict where large populated villages had to be divided into census blocks.

#### Data and sample collection

People living in the house, eating from the same kitchen, and residing in the study area for at least 6 months were defined as household members and were included in the study. Socio-demographic information and medical history including fever episodes in the last 24 hours and in the last month were recorded from all the members through a standardized questionnaire. Physical examination including weight, height, mid-upper arm circumference, axillary temperature and blood pressure were assessed immediately after completion of the questionnaire. Venous blood from one adult and 200μl capillary blood collected in a Microtainer™ from the remaining members of each household were collected and used for blood film examination, haemoglobin measurement, G6PD testing, and molecular analysis. Participants with a positive blood smear for *Plasmodium spp*. were treated with DHP. Those with anaemia were given iron supplementation according to local protocols.

### Laboratory Methods

Parasite species was assessed from Giemsa-stained thick blood films. Peripheral parasitaemia was determined from the number of parasites per 200 white blood cells, assuming a white cell count of 7,300 cells/μL. All positive films and 10% of the negative slides were cross-checked by a second microscopist at the Eijkman Institute for Molecular Biology in Jakarta and discrepancies reviewed by two expert microscopists for final assessment. Haemoglobin levels were determined using a calibrated portable HemoCue® machine (Ångelholm, Sweden). G6PD status was assessed using the G-6-PDH Deficiency Screen by Spot Test Kit® (Trinity Biotech).

Genomic DNA (gDNA) was extracted from ≥50 μL packed red blood cell (RBC) pellets using the QIAamp 96 DNA Blood Kit (Qiagen). *Plasmodium* species confirmation was undertaken in duplicate with 2 μL gDNA template using a nested PCR protocol as described elsewhere [22]. *P*. *falciparum*, *P*. *vivax*, *P*. *malariae*, and *P*. *ovale* small-subunit rRNA DNA clones (MRA-177, MRA-178, MRA-179, and MRA-180; ATCC, Manassas, VA) were used as positive-control materials. The limit of detection (LOD) of the assay was 0.2–2 parasites/μL, as evaluated by using well characterized samples of *P*. *falciparum* and *P*. *vivax* (Piera *et al*. manuscript in preparation). If a duplicate result was discrepant, the assay was repeated. A sample was positive when 2/4 replicates were positive. In case of a 1/4 positivity rate, the assay was repeated a third time and the sample categorized as positive if 2/6 total replicates were positive, but negative if 1/6 replicates were positive.

### Definitions used in the study

An individual was diagnosed as aparasitaemic if both the microscopy and PCR result were negative. Microscopic (patent) parasitaemia was defined in those participants with positive microscopy irrespective of PCR confirmation, assuming that these patients would be detected at a clinical encounter. Submicroscopic (subpatent) parasitaemia was defined in participants who were microscopy negative, but positive PCR result. Where there was discrepancy between the species determined by microscopy and PCR, the diagnosis was classified according to the PCR result. Symptomatic malaria was defined as a positive malaria result by microscopy or PCR associated with a fever or history of fever in the last 24 hours.

Anaemia was diagnosed according to WHO guidelines, defined as a haemoglobin (Hb) concentration less than 11 g/dL in children under 5 years old or pregnant women, less than 11.5 g/dL in children between 5–11 years, less than 12 g/dL in children between 12–14 years old or adult women (>15years) and less than 13 g/dL in adult men. Moderate and severe anaemia was diagnosed when the Hb concentration was less than 10g/dL and 7g/dL in children under 5 years old or pregnant women and less than 11g/dL and 8g/dL respectively for the remaining groups. The remaining cases of anaemia were classified as mild [23].

### Statistical Analyses

Questionnaire and laboratory data were entered into EpiData 3.02 software (EpiData Association, Odense, Denmark). Data were analysed using SPSS v.20.0 for windows software (IBM SPSS Statistics) and STATA 14.1 software (Stata Corp, College Station, TX). Normally distributed data were compared by Student’s t-test and data not conforming to a normal distribution compared by the Mann-Whitney U test. Categorical data were compared by chi-squared test with Yates' correction, or by Fisher's exact test. A multivariable logistic regression model (with robust standard errors to account for clustering by the 16 villages and within household) was used to determine adjusted odds ratios for risk factors associated with anaemia and subpatency. To identify risk factors for the multivariable logistic regression model, variables associated significantly with anaemia or subpatency in a univariable analysis were entered into the equation and the model constructed by a forward stepwise analysis. Analyses for the outcome subpatency were restricted to those individuals with detectable parasitaemia. Multiple fractional polynomial models were used to illustrate the nonlinear association between age and the log odds of detectable parasitaemia, subpatency and anaemia.

### Ethics

All biological samples and patient data were collected with written, informed consent from the patient or a parent or guardian. Ethical approval for this study and the process of informed consent, was obtained from the Eijkman Institute Research Ethics Commission, Eijkman Institute for Molecular Biology, Jakarta, Indonesia, (Ref: KE/FK/763/EC) and the Human Research Ethics Committee of the Northern Territory (NT) Department of Health & Families and Menzies School of Health Research, Darwin, Australia (Ref: HREC-2010-1434).

## Results

Between April and July 2013, 800 households were surveyed comprising 4,010 individuals of whom 2,830 (70.6%) were present at the time of interview. Absentee individuals (n = 1180) were more likely to be adult males 52.3%, (617) or school age children 5–15 years 25.7%, (304). In total, 2,796 of 2,830 (99.8%) participants underwent blood film examination (S1 Fig). Twenty one infants under 5 year old, 2 children aged 5 to 15 years, 3 adults and another with unknown age declined finger prick sampling. The characteristics of household individuals present and sampled during the survey and those absent from the household are presented in S1 Table.

### Microscopy and PCR Confirmation of Parasitaemia

**Fig 1:**
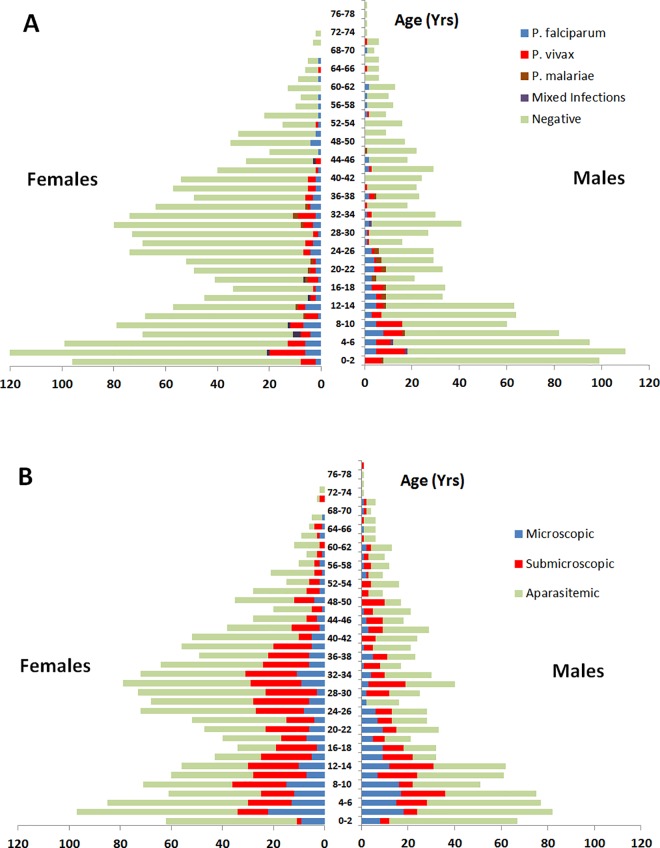
Age stratified prevalence of malaria diagnosed by microscopy and PCR. Microscopy (A) and microscopy and PCR (B).

The age distribution of the 2,796 participants peaked in infancy and early adulthood (Fig 1A), with 59.2% (1654) of the individuals females and 45.6% (1,275) indigenous Papuans (19.2% (536) Highlanders and 26.4% (739) Lowlanders).

Peripheral asexual parasitaemia was diagnosed by microscopy in 12.2% (340) of participants, with *P*. *falciparum* mono-infection present in 145 (5.2%), *P*. *vivax* in 160 (5.7%), *P*. *malariae* in 24 (0.9%) and mixed species infections in 11 (0.4%; Table 1). Gametocytes were present in 2.2% (61/2,796) of individuals; with 88.9% (8/9) of *P*. *vivax* gametocytes present with asexual stages, compared to only 66.7% (32/48) of *P*. *falciparum* gametocytes (*p*<0.001). The geometric mean asexual parasitaemia was 738 parasites/μL [95% CI: 543–1,001] for *P*. *falciparum* and 459 parasites/μL [95% CI: 360–586] for *P*. *vivax*. Fever during the last month was reported in 5.5% (8/145) of those with *P*. *falciparum*, 5.0% (8/160) of those with *P*. *vivax*, and 3.8% (94/2,456) of those negative by microscopy (Table 1).

**Table 1:** Malaria diagnosis and demographic features of survey participants^a^

	Sex		Pregnancy Status ^b^		Age-Group (Years)				Ethnicity			All
n (%)	Male	Female	Non-Pregnant	Pregnant	<1	1–5	5–15	>15	HighlandPapuan	Lowland Papuan	Non-Papuan	
**Microscopic Parasitaemia (Blood Film Positive)** ^**c**^												
***P*. *falciparum***	78 (7.6)	88 (5.7)	48 (4.9)	3 (6.7)	2 (4.3)	19 (5.4)	57 (9.5)	88 (5.6)	40 (8.1)	74 (10.7)	52 (3.8)	166 (6.5)
***P*. *vivax***	76 (7.4)	88 (5.7)	38 (3.9)	2 (4.4)	4 (8.7)	43 (12.2)	53 (8.8)	64 (4.1)	26 (5.3)	51 (7.4)	87 (6.3)	164 (6.4)
***P*. *malariae***	13 (1.3)	7 (0.5)	5 (0.5)	0 (0.0)	0 (0.0)	1 (0.3)	3 (0.5)	16 (1.0)	8 (1.6)	6 (0.9)	6 (0.4)	20 (0.8)
**Mixed species**	3 (0.3)	4 (0.3)	1 (0.1)	0 (0.0)	0 (0.0)	2 (0.6)	3 (0.5)	2 (0.1)	3 (0.6)	4 (0.6)	0 (0.0)	7 (0.3)
**Submicroscopic Parasitaemia (Blood Film Negative and PCR Positive)**												
***P*. *falciparum***	92 (8.9)	138 (9.0)	91 (9.3)	6 (13.3)	0 (0.0)	11 (3.1)	55 (9.2)	164 (10.5)	74 (14.9)	75 (10.9)	81 (5.9)	230 (9.0)
***P*. *vivax***	114 (11)	193 (12.6)	129 (13.2)	2 (4.4)	3 (6.5)	25 (7.1)	89 (14.8)	190 (12.1)	55 (11.1)	98 (14.2)	154 (11.1)	307 (12.0)
***P*. *ovale***	0 (0.00)	1 (0.07)	0 (0.0)	0 (0.0)	0 (0.0)	0 (0.0)	1 (0.1)	0 (0.0)	1 (0.2)	0 (0.0)	0 (0.0)	1 (0.04)
***P*. *malariae***	4 (0.4)	12 (0.8)	11 (1.1)	0 (0.0)	0 (0.0)	0 0.0)	3 (0.5)	13 (0.8)	9 (1.8)	4 (0.6)	3 (0.2)	16 (0.6)
**Mixed species**	25 (2.4)	32 (2.1)	22 (2.2)	0 (0.0)	0 (0.0)	3 (0.8)	14 (2.3)	40 (2.6)	12 (2.4)	30 (4.4)	15 (1.1)	57 (2.2)
**Blood Film Negative and PCR Negative**												
	628 (60.8)	971 (63.3)	633 (64.7)	32 (71.1)	37 (80.4)	249 (70.5)	322(53.7)	991 (63.2)	267 (53.9)	347 (50.4)	985 (71.2)	1,599 (62.3)
**Total**	1,033 (40.2)	1,534 (59.8)	978 (38.1)	45 (1.8)	46 (1.8)	353 (13.8)	600 (23.4)	1,568 (61.1)	495(19.3)	689 (26.8)	1,383(53.9)	2,567(100)

n = number.

^a^ All survey participants with a blood sample

^b^ 22 adult women without negative PCR result

^c^ Following reclassification of species with PCR result and inclusion of 17 infections with only sexual stages present.

In total, 12.8% (357/2,796) of individuals had parasitaemia (either asexual or sexual stages) detectable by microscopy; Table 1 and S1 Fig. PCR analysis was available in 88.6% (2,476/2,796) of cases, although 91 of the remaining individuals were positive by microscopy and their microscopic parasitaemia categorized accordingly. Species was confirmed by PCR in 88.7% (236/266) individuals with microscopic parasitaemia. In the remaining 30 cases, 11 *P*. *falciparum* infections were re-categorized as *P*. *vivax* by PCR, 10 *P*. *vivax* as *P*. *falciparum*, 4 *P*. *malaria*e as *P*. *falciparum*, 2 mixed infections as *P*. *falciparum* mono-infections, and 2 mixed infections as *P*. *vivax* mono-infections. The remaining individual had *P*. *malariae* detected by microscopy, but was negative by PCR and was categorized as having microscopic *P*. *malariae* with a false-negative PCR result. The characteristics of the 2,567 participants who could be classified by both microscopy and PCR are presented in Table 1 and the age distribution in Fig 1B.

**Fig 2:**
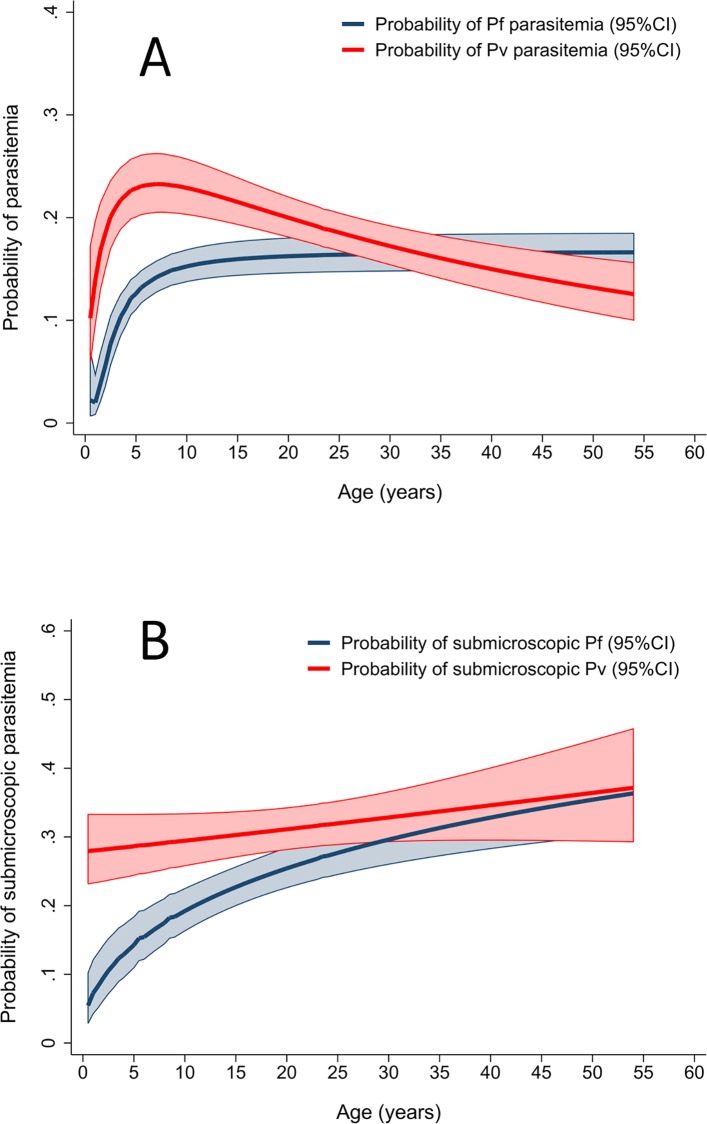
Multiple fractional polynomial curves showing the probability of *P*. *falciparum* and *P*. *vivax* parasitaemia. Probability of parasitaemia by age (A) and probability that detectable *P*. *falciparum* and *P*. *vivax* parasitaemia is submicroscopic by age (B).

In total, 37.7% (968/2,567) individuals had detectable parasitaemia by microscopy or PCR, the prevalence being greatest in children aged 5–15 years (46.3%, 278/600) compared to 28.3% (113/399) in children <5 years, and 36.8% (577/1,568) in adults (*p*<0.001); Table 1, Figs 1 and 2A. The odds of detectable parasitaemia was greater in Papuans compared to Non-Papuans (OR = 2.30 [95%CI: 1.95–2.70]; *p*<0.001). There was no overall difference in the risk of parasitaemia between individuals according to sex, pregnancy, fever in the last month, or bednet ownership. Detectable parasitaemia was significantly less prevalent in the 66 (2.6%) individuals with severe or intermediate G6PD deficiency compared to those who were G6PD normal (OR = 0.44 [95%CI: 0.24–0.80]; *p* = 0.005). The protective effect of G6PD deficiency was more apparent in *P*. *vivax* infection (OR = 0.32 [95%CI: 0.13–0.81]; *p* = 0.010) than in *P*. *falciparum* (OR = 0.46 [95%CI: 0.20–1.08]; *p* = 0.067).

### Risk factors for submicroscopic infections

Risk factors for subpatency were assessed in 968 individuals with detectable parasitaemia, of whom 611 (63%) had submicroscopic infection (Table 1). *P*. *vivax* parasitaemia was more likely to be submicroscopic (65.2%, 307/471) compared to *P*. *falciparum* (58%, 230/396; (OR = 1.35 [95%CI: 1.03–1.78], *p* = 0.038). *P*. *malariae* parasitaemia was submicroscopic in 44% (16/36) of participants.

Overall the risk of submicroscopic infection in those with detectable parasitaemia increased steadily with age (37% (42/113) in children <5, 58% (162/278) in children 5–15 years, and 71% (407/577) in adults; *p*<0.001, Table 2). However, the association between subpatency and age was significant for *P*. *falciparum* (χ2 test for trend, *p*<0.001), but not *P*. *vivax* (*p* = 0.524; Fig 2B). There was a higher risk of submicroscopic infections in females compared to males (OR = 1.5 [95% CI: 1.1–1.9]; *p* = 0.006) and in those who did not own a bednet compared to those who did (OR = 1.4 [95% CI: 1.0–1.8, *p* = 0.025]; Table 2).

**Table 2:** Risk factors for submicroscopic infection in 968 individuals with detectable parasitaemia.

Characteristics	Submicroscopic% (n/total)	Univariable Analysis		Multivariable Analysis	
		Crude OR [95% CI]	*p*	AOR [95% CI]	*p*
**Age**					
**<5 years**	37.2 (42/113)	Reference		Reference	
**5 to 15 years**	58.3 (162/278)	2.36 [1.51–3.70]	<0.001	2.35 [1.6–3.5]	<0.001
**>15 years**	70.5 (407/577)	4.04 [2.65–6.17]	<0.001	3.82 [2.2–6.7]	<0.001
**Gender**	** **	** **	** **	** **	** **
**Male**	58 (235/405)	Reference		Reference	
**Female**	66.8 (376/563)	1.5 [1.1–1.9]	0.006	1.41 [1.1–1.8]	0.002
**Ethnicity**	** **	** **	** **	** **	** **
**Non-Papuan**	63.6 (253/398)	Reference			
**Highland Papuan**	66.2 (151/228)	0.9 [0.6–1.2]	0.544		
**Lowland Papuan**	60.5 (207/342)	1.1 [0.8–1.5]	0.404		
**Bednet use**	** **	** **	** **	** **	** **
**Yes**	58.3 (197/338)	Reference		Reference	
**No**	65.7 (414/630)	1.4 [1.0–1.8]	0.025	1.4[0.9–2.1]	0.199
**G6PD status** ^**a**^	** **	** **	** **	** **	** **
**Normal**	63.3 (602/951)	Reference			
**Deficient**	64.3 (9/14)	1.0 [0.3–2.9]	1		
**Fever in the last 24hr**					
**Yes**	34.4 (11/32)	Reference		Reference	
**No**	64.1 (600/936)	3.4 [1.6–7.1]	0.001	3.2 [1.6–6.5]	0.001
**Fever in last month **					
**Yes**	52.6% (20/38)	Reference			
**No**	63.5% (591/930)	1.6 [0.8–3.0]	0.117		
**Pregnancy** ^b^	** **	** **	** **	** **	** **	** **
**Non Pregnant**	73.3 (253/345)	Reference				
**Pregnant**	61.5 (8/13)	0.58 [0.2–1.8]	0.35			

Abbreviations: CI = confidence interval; OR = odds ratio; AOR = adjusted odds ratio; n = number.

^a^ Data missing on 3 cases

^b^ In 358 adult women.

Fever during the preceding month was reported by in 4% (103/2567) of participants. Only 3.3% (32/968) of individuals with detectable parasitaemia reported fever in the preceding 24 hours, of whom the parasitaemia was microscopic in 65.6% (21/32) and submicroscopic in 34.4% (11/32) (*p* = 0.001). Those with a fever or history of fever in the preceding 24 hours were more likely to be parasitaemic than those without parasitaemia (OR: 2.8 [95%CI: 1.6–5.0]; *p*<0.001); Table 2.

### Risk factors for anaemia

**Fig 3:**
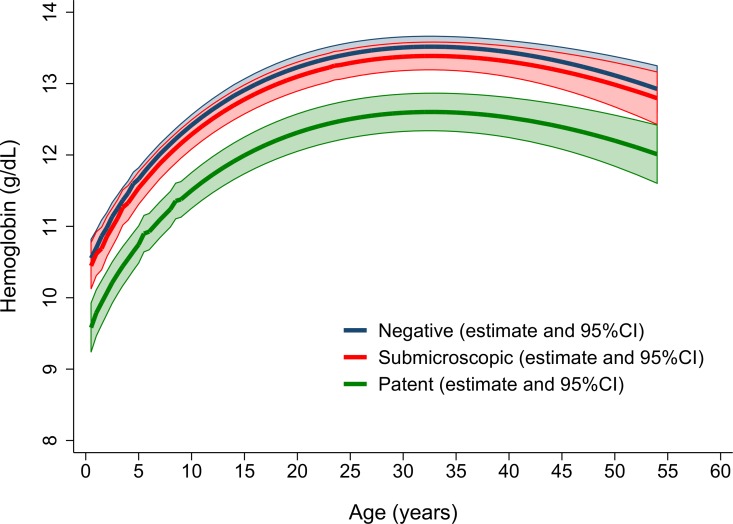
Multiple fractional polynomial curves showing the haemoglobin concentration according to microscopic and submicroscopic parasitaemia and age.

In total haemoglobin concentration was measured in 2757 individuals, of whom 2564 (93.0%) also had documentation of both microcscopic and submicroscopic parasitaemia. In 193 cases no sample was collected for PCR. Haemoglobin (Hb) concentrations varied significantly with gender, age, ethnicity, G6PD status, and malaria species (Table 3 and Fig 3). Anaemia was present in 32.8% (904/2757) of the participants and categorized as mild in 13.1% (365), moderate in 15.8% (443) and severe in 3.4% (96) (S2 Table). The risk of anaemia was greater in children compared to adults (OR = 1.4 [95% CI: 1.2–1.7]; *p*<0.001), Papuans compared to Non-Papuans (OR = 4.05 [95% CI: 3.4–4.8]; *p*<0.001), and females compared to males (OR = 1.18 [95% CI: 1.0–1.4]; *p =* 0.039) (Table 3).

**Table 3:** Haemoglobin concentration of 2757 individuals according to risk groups.

Risk factors	Total	Mean Hb g/dl	Anaemic % (n)	Univariable Analysis	*p*
		(SD)		Crude OR [95% CI]	
**Age (years)**					
<5	497	11.2(2.0)	42.5 (211)	Reference	
5-15y	661	12.1(2.17)	33.3 (220)	0.7 [0.5–0.9]	0.002
>15	1599	13.2(2.62)	29.6 (473)	0.6 [0.5–0.7]	<0.001
**Gender**					
Male	1123	12.9(2.7)	30.5 (343)	Reference	0.039
Female	1634	12.3(2.42)	34.3 (561)	1.18 [1.0–1.4]	
**Ethnicity**					
Non-Papuan	1501	13.3(2.2)	19.2 (288)	Reference	
Highland Papuan	529	11.6(3.0)	52.7 (279)	4.70[3.8–5.8]	<0.001
Lowland Papuan	727	11.9(2.5)	46.4 (337)	3.63[3.0–4.4]	<0.001
**G6PD status**					
Normal	2685	12.6(2.55)	33 (887)	Reference	0.099
Deficient	72	13.5(2.4)	23.6 (17)	0.63 [0.4–1.1]	
**Bednet used**					
Yes	956	12.5(2.6)	35.0 (335)	Reference	0.067
No	1801	12.6(2.5)	31.6 (569)	1.17 [1.0–1.4]	
**Fever in the last 24hr**					
**Yes**	54	12.4(3.6)	42.6 (23)	Reference	0.143
**No**	2703	12.6(2.5)	32.6 (881)	1.5 [0.9–2.6]	
**Fever in last month **					
**Yes**	112	12.3(2.6)	42.0 (47)	Reference	0.040
**No**	2645	12.6(2.5)	32.4 (857)	1.5 [1.0–2.2]	
**Pregnancy**					
Non Pregnant	1000	12.7(2.5)	32.1 (321)	Reference	0.103
Pregnant	45	11.1(2.31)	44.4 (20)	1.69 [0.9–3.1]	
***Plasmodium* species** *					
Negative	1599	12.8 (2.4)	27.6 (442)	Reference	
*P*. *falciparum*	394	11.9 (3.0)	51 (201)	2.7 [2.2–3.4]	<0.001
*P*. *vivax*	470	12.6 (2.6)	32.3 (152)	1.3 [1–1.6]	0.049
*P*. *malariae*	36	11.9 (2.9)	47.2 (17)	2.3 [1.2–4.5]	0.014
*P*. *ovale*	1	9.1	100 (1)	-	-
*Mixed infections*	64	11.9 (3.3)	48.4 (31) (6)	2.5 [1.5–4.1]	0.001

Abbreviations: n = number; Hb = haemoglobin; SD = standard deviation; OR = Odds ratio.

* 193 cases did not have microscopic or PCR diagnosis.

Individuals with detectable parasitaemia were more likely to be anaemic than aparasitaemic individuals (OR = 2.7 [95% CI: 2.1–3.4]; *p*<0.001). In those with microscopic infection, the peripheral parasitaemia of any species was negatively correlated with the haemoglobin concentration (r = -0.184; *p* = 0.001), with a geometric mean parasitaemia of 739 μl^-1^ [95%CI 560–974]) in anaemic individuals compared to 407μl^-1^ [95%CI 327–507] in non-anaemic individuals; *p* = 0.001. Combining microscopic and submicroscopic infections, *P*. *falciparum* was associated with a significantly lower mean Hb concentration (mean = 11.9 g/dL) compared to *P*. *vivax* (mean = 12.6 g/dL); *p =* 0.003), but similar to *P*. *malariae* (mean = 11.9 g/dL) and mixed infections (mean = 11.9 g/dL); Table 3.

**Fig 4:**
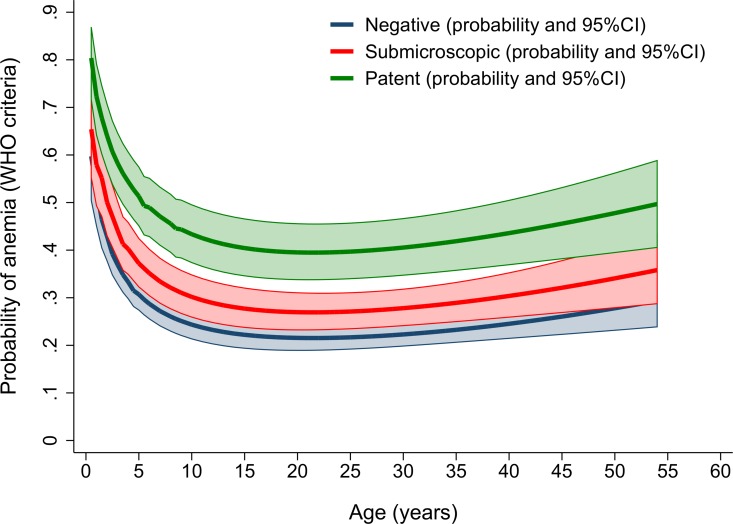
Multiple fractional polynomial curves showing the probability of anaemia by microscopic and submicroscopic parasitaemia and age.

After controlling for age, sex and ethnicity, individuals with either microscopic or submicroscopic *P*. *falciparum* parasitaemia had a greater risk of anaemia compared to aparasitaemic individuals (AOR = 2.9 [95% CI: 1.8–4.6]; *p<*0.001) and 1.9 [95% CI: 1.4–2.7]; *p<*0.001, respectively; Table 4 and Fig 4). However, in those with *P*. *vivax* infections, only those with microscopic parasitaemia had an increased risk of anaemia (AOR = 1.8 [95% CI: 1.1–3.0]; *p =* 0.016). These trends were similar when restricting the analysis to severe or moderate anaemia (S3 Table). The adjusted risks were also similar after excluding 50 individuals with history of fever within the preceding 24 hours (S4 Table).

**Table 4:** Adjusted odds ratios for having anaemia according to WHO standards.

		Age Group (Years)												Overall				
		< 5			5 to 15				> 15									
	n	AOR	95% CI	*p*	AOR	95% CI		*p*	AOR		95% CI		*P*	AOR		95% CI		*p*
**Negative**	1599																	
**Any Microscopic Parasitaemia**^**a**^		4.4	2.2–8.8	<0.001	3.4	1.9–6.0	<0.001		1.6	1.2–2.3		0.002		2.3	1.7–3.2		<0.001	
**Microscopic *P*. *falciparum***	164	8.6	1.8–40	0.006	4.6	2.0–10.2	<0.001		2.0	1.2–3.3		0.005		2.9	1.8–4.6		<0.001	
**Microscopic *P*. *vivax***	163	3.4	1.5–8	0.005	2.3	1.2–4.5	0.018		1.1	0.6–2.2		0.776		1.8	1.1–3.0		0.016	
**Microscopic *P*. *malariae***	20	-	-	-	-	-	-		-	-		-		2.7	[1.3–5.6]		0.007	
**Microscopic Mixed Species**	7	-	-	-	4.0	1.2–13.3	0.025		-	-		-		7.3	1.3–42		0.026	
**Any Submicroscopic Parasitaemia**^**b**^		1.00	0.5–2.1	1	2.1	1.3–3.3	0.002		1.1	0.8–1.4		0.591		1.3	0.9–1.7		0.115	
**Submicroscopic *P*. *falciparum***	230	4.6	1.1–19	0.033	2.9	1.5–5.8	0.002		1.6	1.1–2.2		0.006		1.9	1.4–2.7		<0.001	
**Submicroscopic *P*. *vivax***	307	0.6	0.3–1.2	0.124	1.6	0.9–2.6	0.083		0.7	0.5–1.1		0.090		0.9	0.6–1.2		0.386	
**Submicroscopic *P*. *malariae***	16	-	-	-	-	-	-		-	-		-		1.0	0.3–3.7		0.964	
**Submicroscopic Mixed Species**	57	1.7	0.2–19	0.661	1.8	0.6–5.5	0.307		1.5	0.6–3.6		0.367		1.6	0.7–3.7		0.312	

Abbreviations: n = number; AOR = adjusted odds ratio; CI = confidence interval.

All models control for sex and ethnicity, with robust standard errors to account for clustering within households. In the overall models age group was also included.

^a^3 cases had missing Hb measurement

^b^One submicroscopic case of *P*. *ovale* was not included in the table.

## Discussion

Our study highlights that microscopic blood film examination alone detected only 37% (357/968) of individuals with parasitaemia in this cross sectional survey. Whilst the overall parasite prevalence was greatest in children and Papuan highlanders, the proportion of submicroscopic infections was greatest in older participants, females and those who did not own a bednet. Only 3.3% of participants reported a fever, but asymptomatic microscopic parasitaemia with either *P*. *falciparum* or *P*. *vivax* was associated with a three-fold increased risk of anaemia compared to aparasitaemic individuals. Similar to previous studies of both clinical malaria and asymptomatic infection, the degree of parasitaemia was correlated with anaemia [15, 24]. Whilst these findings are being increasingly recognised and reported, our study strengthens this relationship by demonstrating that the risk of anaemia, including that of moderate severe anaemia, extends to those with submicroscopic parasitaemia, for *P*. *falciparum* but not for *P*. *vivax*.

*Plasmodium* density can be controlled by acquired host immunity; hence, individuals who are repeatedly exposed to malaria have lower parasite densities compared to less-exposed individuals from the same region[25]. In our survey, older participants were almost 4 times more likely to harbor submicroscopic infections than young children who had had less exposure to infection. The odds of submicroscopy was also significantly, albeit marginally, higher in those with *P*. *vivax* parasitaemia versus *P*. *falciparum* infection (OR = 1.35). Similar observations have been reported by other studies in areas of low transmission in the Solomon Islands, Thailand and Myanmar [20, 26] and likely reflect the earlier development of immunity following recurrent *P*. *vivax* infection compared to *P*. *falciparum* [27], as well as the lower peripheral parasitaemias associated with *P*. *vivax* [28]. An increasing body of work emphasizes that the proportion of detectable submicroscopic infections varies hugely, dependent upon the sensitivity of the underlying methodology [5]. Critical factors include the sample volume, collection and storage method, amplification method, and abundance of the target genetic material [20, 29]. Our assay was able to detect peripheral parasitaemias as low as 0.2 parasites/μL, similar to the sensitivity reported from previous studies using 200μl of capillary blood [20]. Individuals with asymptomatic and submicroscopic infections at these levels have been shown to be capable of infecting mosquitoes [9]. Hence, if elimination is to be achieved in a timely manner, suitable strategies are needed to actively identify and treat all infected individuals or populations, rather than passively waiting for symptomatic patients to present to healthcare facilities. Several public health measures have been proposed. Mass screen and test (MST) relies on rapid microscopic diagnosis or rapid diagnostic tests (RDTs) to identify parasitaemic individuals in need of treatment, including both symptomatic and asymptomatic infections [30]. However, in view of the high proportion of submicroscopic infections, MST will eliminate only a fraction of the parasite reservoir; thus its impact on transmission is likely to be limited [31]. Although diagnosis can be improved using more sensitive field-adapted diagnostics such as LAMP [32], the sensitivity of these methods will also still miss the majority of individuals with low level parasitaemia[20].

Alternative strategies are needed to identify individuals and populations at greatest risk of infection using highly sensitive diagnostics so that community-based interventions can then be applied. Interventions such as mass drug administration (MDA) or targeted malaria elimination (TME) have been proposed [31], but these are extremely challenging since they usually require three days of antimalarial treatment administered to individuals who are either unaware that they are infected, or are not infected at all. This has cost implications, as well as exposing apparently well individuals to potential drug-related adverse effects. Rather than personal gain, the individual incentive to comply is, therefore, either altruistic (i.e., for the benefit of the community), or to gain some protection against malaria in the future. Our study provides evidence to mitigate this dilemma, by demonstrating that low-levels infections were associated with a significant morbidity, a three-fold increased risk of anaemia. Individuals with submicroscopic *P*. *falciparum* infection were also at greater risk of anaemia. Although in our study, anaemia was not associated with submicroscopic *P*. *vivax* parasitaemia, this was been reported previously from Brazil [33]. Treatment of patients with asymptomatic microscopic and submicroscopic parasitaemia, therefore, provides potential benefit for both the individual and the community.

Our observations could not be explained by recent symptomatic illness since fever in the preceding month was reported in only 4% (103/2567) of individuals. Rather the anaemia in parasitaemic individuals is likely to have arisen from a combination of low-grade chronic hemolysis, dyserythropoiesis, and reduced iron absorption [34–37]. Interestingly, detectable parasitaemia was significantly less prevalent in individuals with severe or intermediate G6PD deficiency (OR = 0.44), and overall the risk of moderate anaemia was significantly higher in G6PD normal individuals (OR = 6.7 [1.6–28]; *p* = 0.001). Hence, whilst G6PD deficiency increases the risk of oxidative stress and drug-induced haemolysis, this appears to be counterbalanced by its protective effect against malaria-attributable anaemia.

The degree of *P*. *falciparum* associated anaemia is a function of peripheral parasitaemia [24] and this was apparent in our Papuan study with anaemia increasing with higher peripheral parasitaemia. In *P*. *vivax* infections, the level of parasitaemia is significantly lower than that with *P*. *falciparum* and anaemia appears to be related more closely to recurrent and chronic infection [34, 35]. Although we were able to quantify the level of microscopic parasitaemia by microscopy, we did not use a semi-quantitative PCR assay to determine the degree of submicroscopic parasitaemia [26, 38, 39]. It is plausible that in submicroscopic *P*. *vivax* infections the level of parasitaemia, whilst detectable by PCR, was at levels too low to cause significant haemolysis. Our study was also limited by being restricted to a single cross sectional observation with almost 30% of household members absent at the time of the survey. Prospective longitudinal studies will be needed to define better the persistence of submicroscopic infections over time and the associated of with recurrent parasitaemia. Since those individuals absent at the time of the survey were more likely to be adult males with a greater risk of submicroscopic infection, our estimates of parasite prevalence and the proportion of parasitaemia undetected by microscopy are likely to be conservative.

In conclusion, the prevalence of parasitaemia determined by PCR was approximately twice that detected by microscopy. Although most participants in the study were asymptomatic, those with any parasitaemia, including submicroscopic infections, were at significant risk of anaemia. Our analysis identified groups at significantly greater risk of submicroscopic infection, who should be targeted in surveys utilising ultrasensitive diagnostics aiming to quantify hidden reservoirs of infection. The magnitude of submicroscopic infections detected by our studies and others highlights the urgent need to develop new public health strategies and appropriate community-based interventions for the elimination of malaria.

## Supporting Information

S1 Data (CSV)

S1 Fig Study profile. (TIF)

S1 Table Characteristics of household individuals present and sampled during the survey and those absent from the household. (DOCX)

S2 Table The distribution and severity of anaemia. (DOCX)

S3 Table Adjusted odds ratios for severe or moderate anaemia in symptomatic and asymptomatic malaria. (DOCX)

S4 Table Adjusted odds ratios for severe or moderate anaemia in asymptomatic malaria stratified by age group. (DOCX)

## References

[1] World Health Organisation. World Malaria Report: 2014. Geneva:WHO 2015.

[2] Vivax Working G. Targeting vivax malaria in the Asia Pacific: The Asia Pacific Malaria Elimination Network Vivax Working Group. Malaria journal. 2015;14(1):484 10.1186/s12936-015-0958-y 26627892 PMC4667409

[3] Cotter C, Sturrock HJ, Hsiang MS, Liu J, Phillips AA, Hwang J, et al The changing epidemiology of malaria elimination: new strategies for new challenges. Lancet. 2013;382(9895):900–11. 10.1016/S0140-6736(13)60310-4 . 23594387 PMC10583787

[4] Wongsrichanalai C, Barcus MJ, Muth S, Sutamihardja A, Wernsdorfer WH. A review of malaria diagnostic tools: microscopy and rapid diagnostic test (RDT). The American journal of tropical medicine and hygiene. 2007;77(6 Suppl):119–27. . 18165483

[5] Bousema T, Okell L, Felger I, Drakeley C. Asymptomatic malaria infections: detectability, transmissibility and public health relevance. Nature reviews Microbiology. 2014;12(12):833–40. 10.1038/nrmicro3364 . 25329408

[6] Hofmann N, Mwingira F, Shekalaghe S, Robinson LJ, Mueller I, Felger I. Ultra-sensitive detection of Plasmodium falciparum by amplification of multi-copy subtelomeric targets. PLOS medicine. 2015;12(3):e1001788 10.1371/journal.pmed.1001788 25734259 PMC4348198

[7] Imwong M, Hanchana S, Malleret B, Renia L, Day NP, Dondorp A, et al High-throughput ultrasensitive molecular techniques for quantifying low-density malaria parasitemias. Journal of clinical microbiology. 2014;52(9):3303–9. 10.1128/JCM.01057-14 24989601 PMC4313154

[8] Adams M, Joshi SN, Mbambo G, Mu AZ, Roemmich SM, Shrestha B, et al An ultrasensitive reverse transcription polymerase chain reaction assay to detect asymptomatic low-density Plasmodium falciparum and Plasmodium vivax infections in small volume blood samples. Malaria journal. 2015;14(1):520 10.1186/s12936-015-1038-z 26701778 PMC4690410

[9] Okell LC, Bousema T, Griffin JT, Ouedraogo AL, Ghani AC, Drakeley CJ. Factors determining the occurrence of submicroscopic malaria infections and their relevance for control. Nature communications. 2012;3:1237 10.1038/ncomms2241 23212366 PMC3535331

[10] Moreira CM, Abo-Shehada M, Price RN, Drakeley CJ. A systematic review of sub-microscopic Plasmodium vivax infection. Malaria journal. 2015;14(1):360 10.1186/s12936-015-0884-z 26390924 PMC4578340

[11] Cheng Q, Cunningham J, Gatton ML. Systematic review of sub-microscopic P. vivax infections: prevalence and determining factors. PLOS neglected tropical diseases. 2015;9(1):e3413 10.1371/journal.pntd.0003413 25569135 PMC4288718

[12] Roper C, Elhassan IM, Hviid L, Giha H, Richardson W, Babiker H, et al Detection of very low level Plasmodium falciparum infections using the nested polymerase chain reaction and a reassessment of the epidemiology of unstable malaria in Sudan. The American journal of tropical medicine and hygiene. 1996;54(4):325–31. . 8615441 10.4269/ajtmh.1996.54.325

[13] Coleman RE, Kumpitak C, Ponlawat A, Maneechai N, Phunkitchar V, Rachapaew N, et al Infectivity of asymptomatic Plasmodium-infected human populations to Anopheles dirus mosquitoes in western Thailand. Journal of medical entomology. 2004;41(2):201–8. . 15061279 10.1603/0022-2585-41.2.201

[14] Nwakanma D, Kheir A, Sowa M, Dunyo S, Jawara M, Pinder M, et al High gametocyte complexity and mosquito infectivity of Plasmodium falciparum in the Gambia. International journal for parasitology. 2008;38(2):219–27. 10.1016/j.ijpara.2007.07.003 . 17709108

[15] Chen I, Clarke SE, Gosling R, Hamainza B, Killeen G, Magill A, et al "Asymptomatic" Malaria: A Chronic and Debilitating Infection That Should Be Treated. PLOS medicine. 2016;13(1):e1001942 10.1371/journal.pmed.1001942 26783752 PMC4718522

[16] Cottrell G, Moussiliou A, Luty AJ, Cot M, Fievet N, Massougbodji A, et al Submicroscopic Plasmodium falciparum Infections Are Associated With Maternal Anemia, Premature Births, and Low Birth Weight. Clinical infectious diseases: an official publication of the Infectious Diseases Society of America. 2015;60(10):1481–8. 10.1093/cid/civ122 . 25694651

[17] Mohammed AH, Salih MM, Elhassan EM, Mohmmed AA, Elzaki SE, El-Sayed BB, et al Submicroscopic Plasmodium falciparum malaria and low birth weight in an area of unstable malaria transmission in Central Sudan. Malaria journal. 2013;12:172 10.1186/1475-2875-12-172 23714259 PMC3671167

[18] Okell LC, Griffin JT, Kleinschmidt I, Hollingsworth TD, Churcher TS, White MJ, et al The potential contribution of mass treatment to the control of Plasmodium falciparum malaria. PLOS one. 2011;6(5):e20179 10.1371/journal.pone.0020179 21629651 PMC3101232

[19] Mosha JF, Sturrock HJ, Greenhouse B, Greenwood B, Sutherland CJ, Gadalla N, et al Epidemiology of subpatent Plasmodium falciparum infection: implications for detection of hotspots with imperfect diagnostics. Malaria journal. 2013;12:221 10.1186/1475-2875-12-221 23815811 PMC3701503

[20] Imwong M, Nguyen TN, Tripura R, Peto TJ, Lee SJ, Lwin KM, et al The epidemiology of subclinical malaria infections in South-East Asia: findings from cross-sectional surveys in Thailand-Myanmar border areas, Cambodia, and Vietnam. Malaria journal. 2015;14:381 10.1186/s12936-015-0906-x 26424000 PMC4590703

[21] Statistics M. Mimika Regency in Figures. 2014.

[22] Singh B, Bobogare A, Cox-Singh J, Snounou G, Abdullah MS, Rahman HA. A genus- and species-specific nested polymerase chain reaction malaria detection assay for epidemiologic studies. The American journal of tropical medicine and hygiene. 1999;60(4):687–92. . 10348249 10.4269/ajtmh.1999.60.687

[23] WHO. Haemoglobin concentrations for the diagnosis of anaemia and assessment of severity In: System VaMNI, editor. Geneva,: World Health Organization; 2011 p. 6.

[24] Price RN, Simpson JA, Nosten F, Luxemburger C, Hkirjaroen L, ter Kuile F, et al Factors contributing to anemia after uncomplicated falciparum malaria. The American journal of tropical medicine and hygiene. 2001;65(5):614–22. . 11716124 10.4269/ajtmh.2001.65.614 PMC4337986

[25] Doolan DL, Dobano C, Baird JK. Acquired immunity to malaria. Clinical microbiology reviews. 2009;22(1):13–36, Table of Contents. 10.1128/CMR.00025-08 19136431 PMC2620631

[26] Waltmann A, Darcy AW, Harris I, Koepfli C, Lodo J, Vahi V, et al High Rates of Asymptomatic, Sub-microscopic Plasmodium vivax Infection and Disappearing Plasmodium falciparum Malaria in an Area of Low Transmission in Solomon Islands. PLOS neglected tropical diseases. 2015;9(5):e0003758 10.1371/journal.pntd.0003758 25996619 PMC4440702

[27] Michon P, Cole-Tobian JL, Dabod E, Schoepflin S, Igu J, Susapu M, et al The risk of malarial infections and disease in Papua New Guinean children. The American journal of tropical medicine and hygiene. 2007;76(6):997–1008. . 17556601 PMC3740942

[28] Anstey NM, Douglas NM, Poespoprodjo JR, Price RN. Plasmodium vivax: Clinical Spectrum, Risk Factors and Pathogenesis. Advances in parasitology. 2012;80:151–201. 10.1016/B978-0-12-397900-1.00003-7 . 23199488

[29] Wampfler R, Mwingira F, Javati S, Robinson L, Betuela I, Siba P, et al Strategies for detection of Plasmodium species gametocytes. PLOS one. 2013;8(9):e76316 10.1371/journal.pone.0076316 24312682 PMC3848260

[30] Tiono AB, Ouedraogo A, Ogutu B, Diarra A, Coulibaly S, Gansane A, et al A controlled, parallel, cluster-randomized trial of community-wide screening and treatment of asymptomatic carriers of Plasmodium falciparum in Burkina Faso. Malaria journal. 2013;12:79 10.1186/1475-2875-12-79 23442748 PMC3599538

[31] von Seidlein L, Dondorp A. Fighting fire with fire: mass antimalarial drug administrations in an era of antimalarial resistance. Expert review of anti-infective therapy. 2015;13(6):715–30. 10.1586/14787210.2015.1031744 . 25831482

[32] Britton S, Cheng Q, Sutherland CJ, McCarthy JS. A simple, high-throughput, colourimetric, field applicable loop-mediated isothermal amplification (HtLAMP) assay for malaria elimination. Malaria journal. 2015;14:335 10.1186/s12936-015-0848-3 26315027 PMC4552465

[33] Ladeia-Andrade S, Ferreira MU, de Carvalho ME, Curado I, Coura JR. Age-dependent acquisition of protective immunity to malaria in riverine populations of the Amazon Basin of Brazil. The American journal of tropical medicine and hygiene. 2009;80(3):452–9. . 19270298

[34] Douglas NM, Anstey NM, Buffet PA, Poespoprodjo JR, Yeo TW, White NJ, et al The anaemia of Plasmodium vivax malaria. Malaria journal. 2012;11(1):135 Epub 2012/05/01. 1475-2875-11-135 [pii] 10.1186/1475-2875-11-135 . 22540175 PMC3438072

[35] Douglas NM, Lampah DA, Kenangalem E, Simpson JA, Poespoprodjo JR, Sugiarto P, et al Major burden of severe anemia from non-falciparum malaria species in southern papua: a hospital-based surveillance study. PLOS medicine. 2013;10(12):e1001575 10.1371/journal.pmed.1001575 24358031 PMC3866090

[36] Howard CT, McKakpo US, Quakyi IA, Bosompem KM, Addison EA, Sun K, et al Relationship of hepcidin with parasitemia and anemia among patients with uncomplicated Plasmodium falciparum malaria in Ghana. The American journal of tropical medicine and hygiene. 2007;77(4):623–6. . 17978060

[37] Wickramasinghe SN, Abdalla SH. Blood and bone marrow changes in malaria. Bailliere's best practice & research Clinical haematology. 2000;13(2):277–99. 10.1053/beha.1999.0072 . 10942626

[38] Robinson LJ, Wampfler R, Betuela I, Karl S, White MT, Li Wai Suen CS, et al Strategies for Understanding and Reducing the Plasmodium vivax and Plasmodium ovale Hypnozoite Reservoir in Papua New Guinean Children: A Randomised Placebo-Controlled Trial and Mathematical Model. PLOS medicine. 2015;12(10):e1001891 10.1371/journal.pmed.1001891 . 26505753 PMC4624431

[39] Barbosa S, Gozze AB, Lima NF, Batista CL, Bastos Mda S, Nicolete VC, et al Epidemiology of disappearing Plasmodium vivax malaria: a case study in rural Amazonia. PLOS neglected tropical diseases. 2014;8(8):e3109 10.1371/journal.pntd.0003109 25166263 PMC4148206

