# Supplementary figures and images for: Submicroscopic and Asymptomatic Plasmodium Parasitaemia Associated with Significant Risk of Anaemia in Papua, Indonesia
Source: PLoS One. 2016 Oct 27;11(10):e0165340. doi: 10.1371/journal.pone.0165340 (PMC5082812; doi:10.1371/journal.pone.0165340)

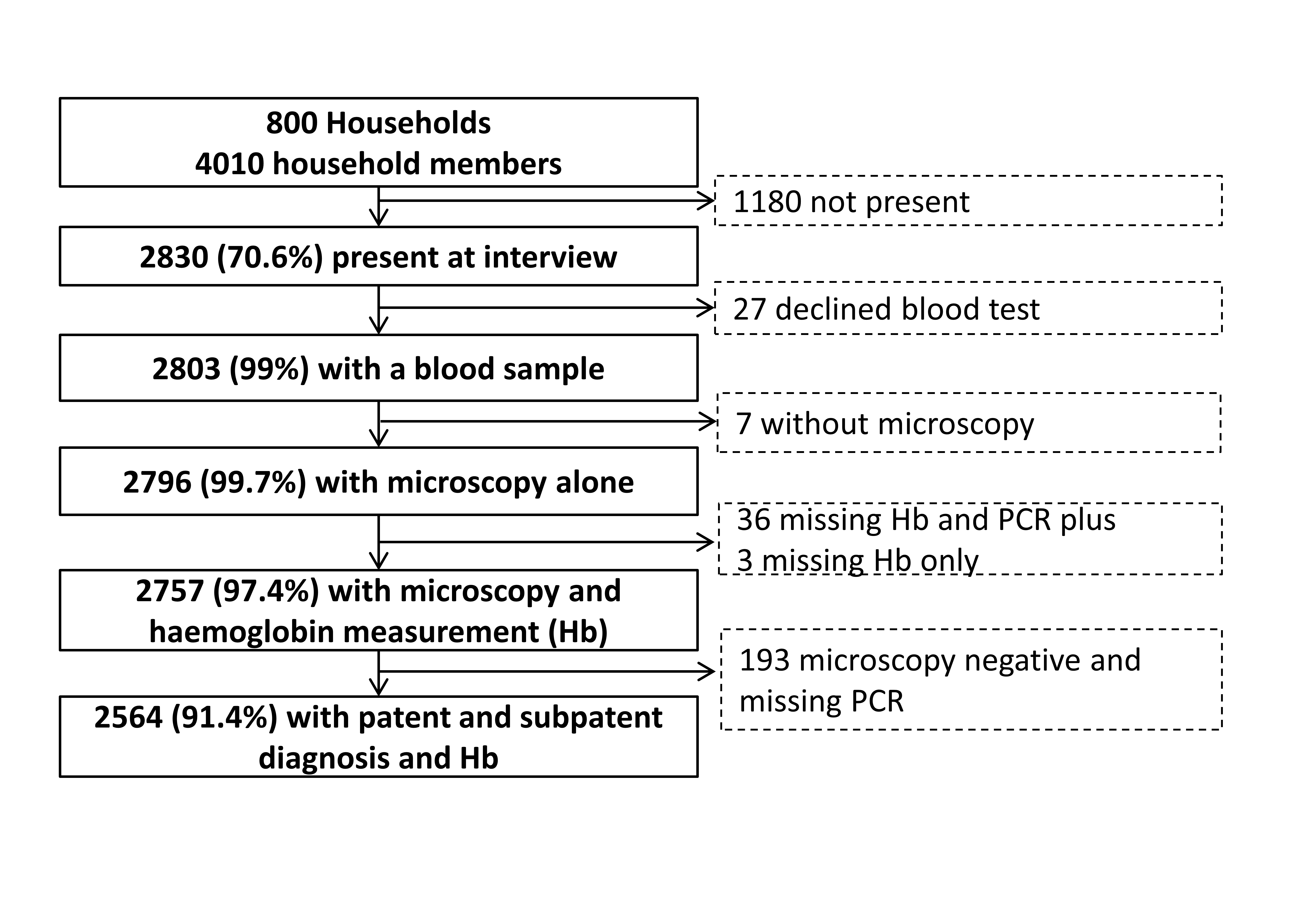

Supplement: S1 Fig — (TIF) [file pone.0165340.s002.tif]
