# Supplementary material for: Submicroscopic and Asymptomatic Plasmodium Parasitaemia Associated with Significant Risk of Anaemia in Papua, Indonesia
Source: PLoS One. 2016 Oct 27;11(10):e0165340. doi: 10.1371/journal.pone.0165340 (PMC5082812; doi:10.1371/journal.pone.0165340)
Supplement: S1 Table — (DOCX) [file pone.0165340.s003.docx]

**S1 Table. Characteristics of household individuals present and sampled during the survey and those absent from the household**

| **Risk factors** | **Present and assessed** | **Absent and not assessed** |  |
| --- | --- | --- | --- |
|  | **% (n)** | **% (n)** | **P value** |
| **Age^#^** |  |  |  |
| <5 years | 19.8%(559) | 6.0% (70) | <0.001 |
| 5-15 years | 23.5% (666) | 25.9% (304) |  |
| >15 years | 56.7% (1604) | 68.1% (800) |  |
| **Gender** |  |  |  |
| Male | 41.1% (1162) | 68.9% (813) | <0.001 |
| Female | 58.9% (1668) | 31.1% (367) |  |
| **Ethnicity** |  |  |  |
| Non-Papuan | 54.4% (1540) | 59.2% (698) |  |
| Highland Papuan | 19.0% (539) | 16.4% (194) | 0.019 |
| Lowland Papuan | 26.5% (751) | 24.4% (288) |  |
| **Fever in the last month** | | | |
| No | 95.9% (2715) | 98.6% (1164) | <0.001 |
| Yes | 4.1% (115) | 1.4% (16) |  |
| Total | 2,830 | 1,180 |  |

^#^7 participants did not have their age documented (1 present and 6 absent at the time of the survey)
