# Supplementary material for: Submicroscopic and Asymptomatic Plasmodium Parasitaemia Associated with Significant Risk of Anaemia in Papua, Indonesia
Source: PLoS One. 2016 Oct 27;11(10):e0165340. doi: 10.1371/journal.pone.0165340 (PMC5082812; doi:10.1371/journal.pone.0165340)
Supplement: S2 Table — (DOCX) [file pone.0165340.s004.docx]

**S2 Table. The distribution and severity of anaemia**

| **Risk factors** | **Non anaemic** | **Mild Anaemia** | **Moderate Anaemia** | **Severe Anaemia** |
| --- | --- | --- | --- | --- |
|  | **% (n/N)** | **% (n/N)** | **% (n/N)** | **% (n/N)** |
| **Age** |  |  |  |  |
| <5 years | 15.4 (286/1853) | 24.4 (89/365) | 23.9 (106/443) | 16.7 (16/96) |
| 5-15 years | 23.8 (441/1853) | 15.1 (55/365) | 31.4 (139/443) | 27.1 (26/96) |
| >15 years | 60.8 (1126/1853) | 60.5 (221/365) | 44.7 (198/443) | 56.3 (54/96) |
| **Gender** |  |  |  |  |
| Male | 42.1 (780/1853) | 38.1 (139/365) | 37.7 (167/443) | 38.5 (37/96) |
| Female | 57.9 (1073/1853) | 61.9 (226/365) | 62.3 (276/443) | 61.5 (59/96) |
| **Ethnicity** |  |  |  |  |
| Non-Papuan | 13.5 (1213/1853) | 18.1 (169/365) | 37.5 (108/443) | 49 (11/96) |
| Highland Papuan | 21 (250/1853) | 35.6 (66/365) | 38.1 (166/443) | 39.6 (47/96) |
| Lowland Papuan | 65.5 (390/1853) | 46.3 (130/365) | 24.4 (169/443) | 11.5 (38/96) |
| **G6PD status ^1^** |  |  |  |  |
| Normal | 97 (1798/1853) | 95.9 (350/365) | 99.5 (441/443) | 100 (96/96) |
| Deficient | 3 (55/1853) | 4.1 (15/365) | 0.5 (2/443) | 0 (0/96) |
| ***Plasmodium species*** | | | | |
| Negative | 67.3 (1157/1853) | 61.9 (208/365) | 47.2 (197/443) | 40.7 (37/96) |
| *P. falciparum* | 11.2 (193/1853) | 16.7 (56/365) | 28.8 (120/443) | 27.5 (25/96) |
| *P. vivax* | 18.5 (318/1853) | 17.6 (59/365) | 18 (75/443) | 19.8 (18/96) |
| *P. ovale* | 0 (0/1853) | 0 (0/365) | 0.2 (1/443) | 0 (0/96) |
| *P. malariae* | 1.1 (19/1853) | 0.9 (3/365) | 2.4 (10/443) | 4.4 (4/96) |
| Mixed infections | 1.9 (33/1853) | 3 (10/365) | 3.4 (14/443) | 7.7 (7/96) |
| **Pregnancy**^2^ |  |  |  |  |
| Non Pregnant | 96.4 (679/704) | 93.8 (135/144) | 94.1 (144/153) | 95.5 (42/44) |
| Pregnant | 3.6 (25/704) | 6.3 (9/144) | 5.9 (9/153) | 4.5 (2/44) |

Anaemia was categorized according to the WHO classification

n=number with category; N= number assessed

^1^ Intermediate deficiency (n=36) and deficient (n= 36) were pooled together

^2^ Adult females only (n = 1045)
