# Supplementary material for: Submicroscopic and Asymptomatic Plasmodium Parasitaemia Associated with Significant Risk of Anaemia in Papua, Indonesia
Source: PLoS One. 2016 Oct 27;11(10):e0165340. doi: 10.1371/journal.pone.0165340 (PMC5082812; doi:10.1371/journal.pone.0165340)
Supplement: S3 Table — (DOCX) [file pone.0165340.s005.docx]

**S3 Table. Adjusted odds ratios for severe or moderate anaemia in symptomatic and asymptomatic malaria.**

|  |  | | | |
| --- | --- | --- | --- | --- |
|  |  |  |  |  |
|  | **n** | **AOR** | **95% CI** | ***p*** |
| **All Symptomatic Parasitaemia** | 17 | 2.7 | 1.5 - 4.7 | 0.001 |
| **Symptomatic *P. falciparum*** | 18 | 5.0 | 2.0 - 13 | 0.005 |
| **Symptomatic *P. vivax*** | 10 | 0.9 | 0.4 - 2.3 | 0.867 |
| **Symptomatic *P. malariae***  **Symptomatic Mixed Species** | 2 | - | - | - |
|  | 2 | - | - | - |
| **All Asymptomatic Parasitaemia** | 911 | 1.5 | 1.1 - 1.8 | 0.003 |
| **Asymptomatic *P. falciparum*** | 376 | 2.0 | 1.5 - 2.7 | <0.001 |
| **Asymptomatic *P. vivax*** | 460 | 1.1 | 0.8 - 1.5 | 0.619 |
| **Asymptomatic *P. ovale*** | 1 | - | - | - |
| **Asymptomatic *P. malariae*** | 34 | 1.4 | 0.6 - 3.1 | 0.386 |
| **Asymptomatic Mixed Species** | 62 | 1.8 | 0.8 - 3.9 | 0.150 |

n=number of participants in the model; AOR = adjusted odds ratio; CI = confidence interval

Multivariate models included: sex, ethnicity and age group.
