# Supplementary material for: Submicroscopic and Asymptomatic Plasmodium Parasitaemia Associated with Significant Risk of Anaemia in Papua, Indonesia
Source: PLoS One. 2016 Oct 27;11(10):e0165340. doi: 10.1371/journal.pone.0165340 (PMC5082812; doi:10.1371/journal.pone.0165340)
Supplement: S4 Table — (DOCX) [file pone.0165340.s006.docx]

**S4 Table. Adjusted odds ratios for severe or moderate anaemia in asymptomatic malaria stratified by age group.**

|  | **Age Group (Years)** | | | | | | | | | | | | **Overall** | | | |
| --- | --- | --- | --- | --- | --- | --- | --- | --- | --- | --- | --- | --- | --- | --- | --- | --- |
|  | **<5** | | | | **5 to 15** | | | | **>15** | | | |  |  |  |  |
|  | n | AOR | 95% CI | *p* | n | AOR | 95% CI | *p* | n | AOR | 95% CI | *p* | n | AOR | 95% CI | *p* |
| **Any Microscopic** | 68 | 4.9 | 2.4-10 | <0.001 | 116 | 3.1 | 1.7-5.6 | <0.001 | 170 | 1.8 | 1.0-3.1 | 0.037 | 354 | 2.6 | 1.8-3.8 | <0.001 |
| **Microscopic *P. falciparum*** | 19 | 10.3 | 2.9-36 | <0.001 | 57 | 4.2 | 1.9-9.3 | <0.001 | 88 | 1.9 | 0.9-4.0 | 0.106 | 164 | 3 | 1.6-5.3 | <0.001 |
| **Microscopic *P. vivax*** | 46 | 3.8 | 1.7-8.6 | 0.001 | 53 | 2 | 1.0-4.0 | 0.051 | 64 | 1.1 | 0.4-2.8 | 0.91 | 163 | 2 | 1.2-3.3 | 0.007 |
| **Microscopic *P. malariae*** | - | - | - | - | - | - | - | - | 2 | - | - | - | 20 | 4.1 | 1.7-10 | 0.002 |
| **Microscopic mixed species** | 2 | 1.8 | 0.1-22 | 0.659 | 3 | 7.8 | 2.6-24 | <0.001 | - | - | - | - | 7 | 6.1 | 3.4-10.7 | <0.001 |
| **Any Submicroscopic** | 42 | 1 | 0.4-2.5 | 0.987 | 162 | 2.3 | 1.2-4.3 | 0.013 | 407 | 1.3 | 0.9-1.8 | 0.135 | 611 | 1.5 | 1.0-2.1 | 0.029 |
| **Submicroscopic *P. falciparum*** | 11 | 5.2 | 1.5-18 | 0.01 | 55 | 3.3 | 1.3-8.4 | 0.015 | 164 | 2 | 1.2-3.4 | 0.007 | 230 | 2.4 | 1.5-3.9 | <0.001 |
| **Submicroscopic *P. vivax*** | 28 | 0.3 | 0.1-1.1 | 0.068 | 89 | 1.7 | 0.9-3.0 | 0.075 | 190 | 0.7 | 0.5-1.1 | 0.123 | 307 | 0.9 | 0.6-1.4 | 0.632 |
| **Submicroscopic *P. malariae*** | 0 | - | - | - | 3 | - | - | - | 12 | - | - | - | 16 | 1.5 | 0.6-4.1 | 0.415 |
| **Submicroscopic mixed species** | 3 | 1.5 | 0.2-10 | 0.652 | 14 | 1.5 | 0.4-6.1 | 0.597 | 40 | 1.9 | 0.8-4.2 | 0.14 | 57 | 1.7 | 0.7-3.8 | 0.223 |
| **Negative** | 286 | Ref |  |  | 322 | Ref |  |  | 991 | Ref |  |  | 1,599 | Ref |  |  |

Abbreviations: n=number; AOR= adjusted odds ratio; CI=confidence interval

Models control for sex and ethnicity with robust standard errors to account for clustering within households. For the overall models age group was also included.
